# Transabdominal laparoscopic retroperitoneal neurectomy for chronic pain after inguinal hernia repair and appendicectomy –a matched-pair study

**DOI:** 10.1186/s12893-017-0282-2

**Published:** 2017-07-20

**Authors:** Ioannis Karampinis, Johannes Weiss, Lothar Pilz, Stefan Post, Florian Herrle

**Affiliations:** 1 Department of Surgery, Mannheim University Medical Centre, University of Heidelberg, Theodor-Kutzer-Ufer 1-3, 68167 Mannheim, Germany; 2 Department of Surgery, GRN-Klinik Schwetzingen, Schwetzingen, Germany; 3 Department of Statistics, Mannheim University Medical Centre, University of Heidelberg, Mannheim, Germany

**Keywords:** Chronic inguinal pain, Neurectomy, Inguinal neuralgia

## Abstract

**Background:**

Chronic debilitating pain is a rare but significant cause of postoperative morbidity after inguinal surgery. Such pain is usually of neuropathic origin and frequently caused by intraoperative nerve damage. In this retrospective matched-pair study we analysed results of a minimal-invasive approach to neurectomy on quality of life and pain relief.

**Methods:**

From March 2010 to January 2012, 9 patients developing chronic neuropathic pain after inguinal hernia repair (8 patients) or open appendicectomy (one patient) were operated using a laparoscopic transabdominal approach in our department. Clinical examinations and specific questionnaires on pain and quality of life (PainDetect, SF-36) were completed 6 months to 3 years after neurectomy. Every patient was matched with one patient without chronic pain.

**Results:**

Seven of nine patients had severe or very severe pain before neurectomy, two had mild pain but refused a conservative treatment. Four patients were free of pain after neurectomy, three described an improved pain status, whereas two did not observe any change in pain. Within a follow-up period of 14,3 months, no deterioration of pain or other complications were observed. Patients who underwent neurectomy had significantly lower quality of life compared to the control group. No postoperative complications were observed.

**Conclusions:**

Laparoscopic transabdominal neurectomy represents a possible surgical approach in treating patients with chronic disabling postoperative groin pain requiring surgery. This technique was feasible, safe, and effective in our series to relieve chronic debilitating pain in the majority of our patients with comparable results to other published approaches.

## Background

Chronic groin neuralgia is a well-known complication of operations involving a transverse incision of the right lower abdominal wall and occurs in approximately 0,5–6% of the cases postoperatively [1]. Inguinal hernia repair and open appendicectomy represent the main part of such operations. Chronic postoperative pain, as defined by duration longer than 3 months [2, 3] is often difficult to differentiate, mainly because of the complex innervation of the anatomic region, and is classified according to the pathophysiological origin in neuropathic and nociceptive pain. Neuropathic pain is commonly the result of intraoperative damage of one of the four nerves innervating the groin, the iliohypogastric, ilioinguinal, genitofemoral, and lateral femoral cutaneous nerve [4].

Nociceptive pain evolves as a result of tissue reaction to the inflammatory process caused by the operation, mesh related fibrosis or post-operative fibrosis [5]. Neuropathic pain on the other side is a result of direct or indirect damage of the local nerves caused by transection, dissection or entrapment of the nerves in suture material [5]. This can occasionally lead to traumatic neuroma formation. The differential diagnosis of these two types of pain still remains challenging. MR (magnet resonance) neurography has been suggested by Amid as a useful radiology test for neuropathic pain, but the main diagnostic tool presently is clinical experience [6].

Stulz and Pfeiffer described surgery in 1982 as an option for treating nerve-associated pain [7]. They performed a cicatricotomy to treat inguinal neuralgia after inguinal herniotomy, appendicectomy, and gynaecological operations. They postulated that the nerve most endangered after hernia operations is the ilioinguinal, and after appendicectomy the iliohypogastric. Meanwhile surgical neurectomy constitutes an important instrument in the treatment of chronic neuropathic pain, offering a feasible therapeutic solution when conservative treatment has failed [8–13].

The surgical approach for neurectomy still remains a controversial issue. The traditional standard approach is a two-staged approach: anteriorly to the ilioinguinal and iliohypogastric nerves and from the flank to the lateral femoral cutaneous and genitofemoral nerve [6]. Surgery success rates in reducing chronic pain are variable, mainly depending on the patient selection criteria, and have been reported between 30% and 100% [9, 10]. Furthermore, minimal-invasive retroperitoneal approaches have also been described for accessing the groin nerves offering similar success rates [14, 15].

In this study we evaluated a transabdominal laparoscopic approach for accessing the retroperitoneal space for neurectomy. To our best knowledge, this is the first study to evaluate the impact of surgical therapy on chronic neuropathic pain in a series of patients using this surgical approach and evaluating the quality of life.

## Methods

### Population

We identified all nine patients who had developed chronic groin pain after operations of the right lower abdomen and had undergone a laparoscopic transabdominal retroperitoneal neurectomy (LTRN) in our department between March 2010 and January 2012. The clinical records of those patients were retrospectively evaluated. Patient characteristics are listed on Table 1.

**Table 1:** Patient-characteristics of the LTRN group

	Sex	Primary Operation	Pain treatment prior Nx	Interval prim. OP-Nx (months)	Neurectomied nerve	Interval Nx-follow up (months)	Post Nx hospital stay (days)
1	F	Laparoscopic appendicectomy	Acupuncture, homeopathy, analgesics	33.0	GFN, IIN, LFCN	24	6
2	F	Shouldice	analgesics	122.2	GFN, LFCN	19	5
3	F	Lotheissen- Mc Vay	acupuncture, nerve block, analgesics, local infiltrations	32.2	GFN, IIN, LFCN	14	3
4	M	Anterior herniotomy	nerve infiltrations	139.5	LFCN, FBGFN	14	8
5	M	TEP bil.	analgesics, infiltrations	45.8	GFN, LFCN bilaterally	12	3
6	M	Lichtenstein	no	3.6	GFN, IIN, LFCN	10	1
7	M	Incisional herniotomy	analgesics, psychosomatic	355.1	GFN, LFCN	5	4
8	M	Shouldice	antidepressiva	176.1	GFN, LFCN	5	2
9	M	Lichtenstein	no	7.0	GFN, LFCN	26	1

*OP* Operation, *GFN* genitofemoral nerve, *IIN* ilioinguinal nerve, *LFCN* lateral femoral cutaneous nerve, *FBGFN* femoral branch of the genitofemoral nerve, *Nx* neurectomy, *LTRN* laparoscopic transabdominal retroperitoneal neurectomy, *TEP* total extraperitoneal plastic, *bil* bilaterally

Before LTRN (laparoscopic transabdominal retroperitoneal neurectomy) clinical examination by a specialized hernia surgeon and consultant (J.W.) had been performed in all patients in order to exclude a recurrent hernia. Pubic osteitis, orthopaedic pain syndromes, and other pathologies had been clinically or sonographically ruled out. LTRN had been indicated as a tailored decision combining clinical findings and respecting individual patient preferences. The neurectomy was performed using a transabdominal laparoscopic approach and is described in detail below. All resected nerves were histologically examined.

Ethic approval was granted from our institutional ethics committee prior to the study. Patients agreed in an informed consent their participation to this study as well as the scientific use of their data.

Identified neurectomy patients were invited by phone for a consultation and follow-up examination including a clinical examination. After the clinical examination, all patients were asked to fill out 3 questionnaires: The German version of the self-administered quality of life questionnaire (SF-36) [16], a validated questionnaire for neuropathic pain (Pain-Detect) [17], and a translated questionnaire adapted from a former paper published by Loos et al. [13].

Through the second clinical examination, recurrent hernia or other confusing painful groin pathologies were ruled out. A circumstantial neurological examination was also performed in order to reveal regional sensoric defects.

#### PainDETECT-questionnaire

Freyhagen et al. [17] developed the pain-detect questionnaire to assess whether patient reported pain may be of neuropathic or nociceptive origin. The end-point comes from the evaluation of the summative score and varies from 1 to 38 points with 1–13 referring to a high possibility of nociceptive pain, 13–18 is interpreted as indeterminable zone, and a score > 18–38 corresponds to neuropathic pain with a high probability. The patients were requested to fill out this questionnaire concerning their actual pain status.

#### SF-36

The SF 36 [16] was developed to evaluate the quality of life and is divided into 8 scaled sections, each of them including several questions. The sum scores of each section can be transformed in a 0–100 scale, assuming that all questions are equally weighted. Low scores express disability. The sections tested through this questionnaire are physical functioning (PF), physical role functioning (PRF), bodily pain (BP), general health perceptions (GHP), vitality (V), social role function (SRF), emotional role function (ERF), and mental health (MH).

#### Matched control group

A matched patient group for comparisons of generic quality of life data and neuropathic pain status was constructed using our clinical database records. These patients had undergone similar primary surgery without developing chronic postoperative pain, and were matched according to age, gender, primary operation, and date of the primary operation (categorized by 5-year-intervals). Identified patients of the matched control group were contacted by telephone and requested to fill out the same three questionnaires, which were sent by mail.

### Technical description of transabdominal laparoscopic retroperitoneal neurectomy

A 45-degree vision camera was inserted through a 10 mm trocar using a horizontal infraumbilical incision. Two further 5 mm incisions were performed. The sigmoid colon or caecum and ascending colon were mobilised. The ureter and the testicular vein were identified and preserved. The psoas muscle was visualized and any attachments were carefully removed in order to be able to identify the ilioinguinal and iliohypogastric nerve. These nerves were identified after penetrating the psoas muscle, while travelling on the quadratus lumborum muscle to the lateral abdominal wall. Depending on the patient’s symptoms, a triple-nerve neurectomy of the ilioinguinal, iliohypogastric, and genitofemoral nerve was not routinely performed. We selectively resected those nerves, the innervation region of which corresponded to our clinical symptoms and the localization of the pain. The genitofemoral nerve was resected in case of pain in the genital region, the ilioinguinal nerve in case of pain in the proximal, medial part of the upper thigh. The lateral femoral cutaneous nerve was resected in case of pain in the lateral upper thigh. In cases of overlapping in the proximal thigh we resected both the ilioinguinal and the lateral femoral cutaneous. We did not have any patients with pain directly at the old incision scar corresponding to a possible damage of the iliohypogastric nerve. The genitofemoral nerve was identified on the psoas muscle, lateral and caudal to the ilioinguinal nerve, before it’s splitting. The ends were electrocoagulated in order to prevent the formation of traumatic neuroma, and buried into the internal oblique muscle. Lateral to the genitofemoral nerve we identified the thinner lateral femoral cutaneous nerve.

### Statistics

Sum score calculation and transformation of the quality of life questionnaire SF 36 was performed according to the German handbook of interpretation by Bullinger [18].

Descriptive statistics and exploratory association as well as group comparison analyses were performed with StatXact from Cytel Studios version 9.0 (2012 Cambridge, MA, USA) and SAS version 9.2 (SAS Institute Inc. 2013, Cary, NC, USA). The level of significance was set at *p* < 0.05.

## Results

The mean age of the LTRN- group was 57.1 years (SD 11.8) and the median 60 years (Table 1). The mean age of the control group was 56.8 years (SD 12.9) and the median 58. There was no significant difference in the ages of both groups (*p* = 0.955). Patient 3 had a Lotheissen-Mc Vay operation and was matched with a Shouldice operation. Patient 4 had undergone an anterior herniotomy without mesh and was also matched with a Shouldice operation (Table 2). Patient 7 had undergone an incisional herniotomy without mesh after a conventional appendicectomy.

**Table 2:** Patient-characteristics of the control group

	Gender	Primary Operation	Interval primary Operation-Follow up (months)
1	F	Lap. appendicectomy	40
2	F	Shouldice	95
3	F	Shouldice	94
4	M	Shouldice	164
5	M	TEP	73
6	M	Lichtenstein	107
7	M	Open appendicectomy	159
8	M	Shouldice	106
9	M	Lichtenstein	106

*TEP* Total extraperitoneal hernia repair, *M* male, *F* female, *lap* laparoscopic

All patients were under common analgetic therapy before the neurectomy, 3 had tried unsuccessful nerve blockage and local infiltration, 2 had tried acupuncture and psychosomatic therapies and 5 had tried an additional antidepressive medication. Two patients required the surgery as primary pain-relieving option despite our advice.

The median time interval between primary operation and LTRN was 45.8 months (range 36–355). The mean interval between neurectomy and follow up was 14.3 months (SD 7.5) and the median 14 (range 5–26 months).

Patients 3,4,5,6 and 8 had urologic comorbidities and Patient 2 had orthopedic comorbidities. The other patients did not have a relevant medical history.

### Primary operation

The longitudinal development in the pain status of all patients from just before the primary operation till the end of the follow-up period was acquired using the questionnaire of Loos [13]. Pain status before undergoing the primary operation was significantly different between the two groups: four Patients from the LTRN-group had no or mild pain before the primary operation, whereas 8 patients from the control group had mild or no pain. Five patients from the LTRN- group had severe or very severe pain before the primary operation, whereas only one patient from the control group had very severe pain (*p* = 0.264).

Seven Patients from the LTRN- group had severe or very severe pain after the primary operation and two had mild pain. Eight patients from the control group had mild pain or no pain and only one described moderate pain. The level of pain after the primary operation between the two groups was statistically significant (*p* = 0.0004).

Concerning the complication frequency of the primary operation in the LTRN- group, 3 patients had no complications, 3 had skin numbness, 2 had numbness with hypersensitivity and 1 had numbness, hypersensitivity, and postoperative bleeding which was conservatively treated. In the control group, 5 patients had no complications, 1 developed a wound infection, 2 numbness, and 1 patient developed a bulge (complication frequency between the two groups not significant different *p* = 0,637).

### LTRN

#### Pain status before undergoing LTRN

Seven patients from the LTRN- group had severe or very severe pain before neurectomy, and two had mild pain. None of the patients described a change in the pain character or severity during the period after the primary operation and before the neurectomy. From the two patients who were operated with only having mild pain, the first one had already experienced an anterior neurectomy of the contralateral side with very good results, after several years of conservative treatment and did not wish to try again a prolonged medical therapy. The pain of the second patient was mainly localized in the scrotal region, accompanied with sensory disorders and was resistant to common analgetic therapy. Both patients received a detailed description of the current trends in the treatment of such symptoms and decided to undergo surgery.

#### Pain status and clinical results after LTRN

After LTRN, four patients had no pain, two patients had mild pain, one had moderate pain, one had severe pain, and one had very severe pain (Table 3).

**Table 3:** Pain status of the LTRN-group

	Pain prior hernia-surgery	Pain prior Nx-Surgery	Pain after Nx-Surgery	Pain during sex/orgasmus prior Nx	Pain during sex/orgasmus post-Nx	Repeat of Nx
1	4	2	1	yes	yes	yes
2	4	4	2	no	n.a.	yes
3	5	4	3	n.a.	n.a.	n.s.
4	2	5	5	n.a.	n.a.	no
5	2	4	4	yes	n.a.	n.s.
6	1	4	2	n.a.	n.a.	yes
7	4	5	1	n.a.	n.a.	n.s.
8	5	2	1	yes	no	yes
9	1	4	1	yes	no	yes

1: no pain, 2: mild pain, 3: moderate pain, 4: severe pain, 5: very severe pain

*n.a.* not appropriate, *n.s.* not sure, *LTRN* laparoscopic transabdominal retroperitoneal neurectomy, *Nx* neurectomy

According to the adapted Loos-questionnaire, 4 patients from the LTRN- group described painful events during sex or orgasm before the neurectomy, whereas only one patient from the control group had such complaints (*p* = 0.071). From the four patients with pain during sex, two were completely free of pain after the neurectomy, one described an improvement in the pain status and the last one didn’t notice any change.

Five patients described constant or regularly pain, four had occasionally painful events. Pain localisation was the inguinal region for all patients.

The LTRN- group was retrospectively asked how they would rate the result of the neurectomy on a scale of 1 to 5 (1: excellent, 2: good, 3: moderate, 4: bad, 5: very bad). One rated it as excellent, 3 as good, 2 as moderate and 3 as bad. One patient rated the operation as bad, despite describing a clear improvement in his pain status. All patients were asked if they would decide to do the neurectomy when retrospectively knowing the result. Five answered yes, one answered no, and three were not sure.

Comparing the pain status before and after neurectomy, seven patients described a remission of pain and two a persistence of the symptoms without deterioration of the pain. Two patients who described an improvement of their pain status were not sure if they would undergo the neurectomy again (Table 3). One patient who described the result of the neurectomy as bad would retrospectively decide doing the operation again. One patient classified the result of the neurectomy as moderate whilst declaring a complete remission of pain after the operation.

Concerning the sensory status of the lower abdominal inguinal region and inner thigh after neurectomy, one patient had normal sensibility, seven hypoesthesias and one hyperesthesia. The localisations of the hypoesthesia involved in all cases the ventral thigh, and in one case the scrotum and pubic region (pat. 8). The hyperesthesia involved the proximal thigh (pat. 3). Some patients described a decreasing tendency of sensory symptoms over time. One patient of the LTRN- group was disabled due to the chronic pain (pat. 5).

Two Patients had over 18 points suggesting neuropathic pain, two patients had less than 13 points, and the rest was in the undifferentiated zone. All patients of the control group had less than 13 points with the vast majority of them being at 0 (Table 4).

**Table 4:** PainDETECT® scores: comparison of median values between the LTRN- and the control group

PainDETECT	Median-values		
	LTRN *N* = 9	Control *N* = 9	*p*-Value (t-test)
Question 1	2	0	0.00399
Question 2	5	0	0.00123
Question 3	4	0	0.00185
Question 4	2	0	0.00078
Question 5	1	0	0.05031
Question 6	2	0	0.03147
Question 7	1	0	0.00399
Question 8	0	0	0.22242
Question 9	1	0	0.00399
Question 10	3	0	0.00123
Question 11	2	0	0.01419
Final Score	14	0	0.00029

Source: Freyhagen et al. [17]

LTRN-patients had still significantly lower quality of life after the neurectomy compared with the control group in all 8 scales of the SF-36, except the emotional role function (Table 5). The co-variate factor with the highest influence in developing chronic pain was pain during sex or orgasm (*p* = 0.031). The next factor influencing the development of chronic pain was the young age without reaching a significant level (*p* = 0.07).

**Table 5:** Comparison of SF-36 mean and median values for the LTRN- and the control group

	Median LTRN *N* = 9	Median Control *N* = 9	Mean LTRN-	Mean Control	*p*-Value (t-test)
PF	60	100	58.33 ± 31.97	90.00 ± 18.41	0.0305
PFR	75	100	50.00 ± 45.64	91.67 ± 23.57	0.0406
BP	42	100	46.11 ± 28.48	96.00 ± 11.31	0.0010
GHP	57	77	53.67 ± 19.83	79.67 ± 14.09	0.0091
V	50	70	48.33 ± 23.80	73.89 ± 10.48	0.0179
SRF	50	100	55.56 ± 29.53	91.67 ± 19.54	0.0120
ERF	100	100	74.07 ± 40.91	96.30 ± 10.48	0.1708
MH	44	92	52.89 ± 21.13	88.89 ± 8.39	0.0016

*PF* physical functioning, *PRF* physical role functioning, *BP* bodily pain, *GHP* general health perceptions, *V* vitality, *SRF* social role function, *ERF* emotional role function, *MH* mental health

## Discussion

In this study we performed a retrospective analysis of patients treated with a transabdominal laparoscopic neurectomy for chronic pain after operations of the groin or right lower abdomen. Several therapeutic approaches have been recommended in order to treat chronic postoperative groin pain. To the best of our knowledge, none of the existing studies has evaluated the quality of life of those patients.

Giger used an endoscopic retroperitoneal approach in 39 patients with chronic groin pain, describing complete pain relief in 80% of the cases, a transient relief in 16% [14]. The genital branch of the genitofemoral nerve can, according to Giger, not be found through the anterior approach. Muto used the same approach and performed an ilioinguinal and genitofemoral neurectomy in 6 patients with 100% pain relief and postulated that these two nerves are responsible for the chronic groin pain after groin surgery [15].

Vuilleumier used an open approach to perform a radical IIN and IHN neurectomy after mesh removal without routinely dissecting the GFN, having 95% pain relief in 49 patients [10]. Kline performed a targeted anterior neurectomy after successful injection of local anesthesia having almost 100% success rates [19]. Amid operated 225 patients using an anterior approach and performing a triple neurectomy with 80% success rates 1 month after the operation [6]. Valvekens performed a symptom-orientated treatment with combination of mesh removal with or without neurectomy, having a pain relief in one third of the patients [9]. Loos performed a triple neurectomy with mesh removal in 49 patients with 76% success [13].

In our study 7 of our patients described a clear improvement of their pain, whereas 2 did not report any change. Furthermore, we saw that the quality of life of these patients despite the mildness of the pain is significantly reduced in all aspects compared with the control group except the emotional role function. The SF-36 values of the control group were similar to reference values on normal population according to other published studies [20].

Chronic postoperative pain after surgery of the right lower abdomen and inguinal region is an underestimated problem [21]. Various studies have shown that despite the clinical progress, the number of patients remaining clinically and socially disabled after such a common operation, is relevant and the relieve we can offer to those patients, often inadequate. Prevention of chronic pain is currently receiving crucial attention, mainly in the area of pre-emptive anaesthesia and intraoperative pain management [5, 22]. According to Caroll et al., there is a critical time frame, during which we can influence the development of chronic pain. The intraoperative damage of a nerve still is the main cause of chronic debilitating pain [22]. However, an exact treatment algorithm of nerve damage is not clearly defined, and is usually performed according to the experience of each surgeon. There is no consensus on how and if nerve blocks should be performed, and how the results should be interpreted [23–25].

Surgical treatment clearly plays an important role in the treatment of chronic debilitating neuropathic pain. The surgical approach and the way the neurectomy should be performed, is so far not exactly defined. The standard anterior two-stage approach offers the advantage of recognizing direct damage of the situs, involving nerve entrapment, hematoma or adhesions which could explain chronic pain and simultaneously intervene.

The main disadvantage is that the situs is difficult to explore because of severe adhesions and scars, often resulting in additional damage, wound healing disorders, and longer hospitalisation, without resolving the symptoms.

Furthermore, in the case of neuropathic pain, the nerve may not be resectable proximally to the damage which may lead to persistent pain due to remaining parts of the unresected, damaged nerve or secondary neuroma formation. In our series, all resected nerves were histologically examined. In 4 cases the pathological report revealed nerve fibrosis, although the nerve was resected proximally enough to the suspected area of damage. In one case a traumatic damage with axonal degeneration was discovered. In the other 4 cases the histological report did not reveal any relevant pathological changes of the nerves.

Minimal invasive non-anterior approaches to neurectomy offer the advantage of resecting the nerve proximally enough, in order to achieve pain relief. Further manipulation of the operated and therefore surgically inhostile area is avoided. Laparoscopy offers, according to various publications, similar - if not better results than the anterior approach [14, 15, 26].

The advantage of the transabdominal laparoscopic surgical approach in comparison to the laparoscopic retroperitoneal approach lies on the preservation of the anatomical structures in a way, which is familiar to the surgeon. In addition, it offers the ability of using the already existing scars from old operations, as many of these patients have already undergone a laparoscopy. Through the laparoscopic transabdominal approach, the surgeon gains access to both sides, in case the patient has developed chronic neuropathic pain after a bilateral procedure. Other causes of abdominal pain, such as adhesions, can also be excluded during the laparoscopy.

A possible disadvantage of the method is that local causes of chronic pain, such as meshoma or other inflammatory processes, cannot be ruled out or treated. A staged and careful selection of the patients could avoid such confounding factors. A recent study [27] postulated that the incidence of chronic pain after herniotomy with mesh implantation is similar to the incidence of recurrence after herniotomy without mesh. In the first case, however, we have to take into account the 20% of patients with severe chronic pain who we cannot heal [27], whereas in the second case, we can always try a mesh implantation.

### Limitations

The limitations of our study include the small number of the patients in the LTRN-group and the matching group. The optimal matching group would consist of patients previously treated with medication or intervention. We had initially planned a 1:3 matching, which could not be achieved for all patients, due to the long time interval from the primary operation to the neurectomy (for one patient almost 20 years). Further limitations are the subjectivity of the way in which every patient or matching candidate understood the questions and the bias in the chronological way in which the questions were set, despite the fact that all patients were asked to fill out the questionnaires concerning the pain status at the time of the follow-up. The decision to perform the neurectomy was based on the clinical examination and the experience of the surgeon. Quantifiable techniques such as quantitative sensory testing were not implemented since the experience with this method was not enough at the time we performed the trial.

## Conclusions

Laparoscopic transabdominal neurectomy represents an optimal surgical approach in patients with chronic disabling postoperative groin pain requiring surgical treatment. This technique was feasible, safe and effective in our series to relieve chronic debilitating pain in the majority of our patients with comparable results to other published approaches.

## Abbreviations

FBGFN: Femoral branch of genitofemoral nerve
GBGFN: Genital branch of genitofemoral nerve
GFN: Genitofemoral nerve
IHN: Iliohypogastric nerve
IIN: Ilioinguinal nerve
LFCN: Lateral femoral cutaneous nerve
LTRN: Laparoscopic transabdominal retroperitoneal neurectomy
Nx: Neurectomy
OP: Operation
SD: Standard deviation
TEP: Total extraperitoneal plastic
